# Model-Based Assessment of Estuary Ecosystem Health Using the Latent Health Factor Index, with Application to the Richibucto Estuary

**DOI:** 10.1371/journal.pone.0065697

**Published:** 2013-06-13

**Authors:** Grace S. Chiu, Margaret A. Wu, Lin Lu

**Affiliations:** 1 CSIRO Mathematics, Informatics and Statistics, Commonwealth Scientific and Industrial Research Organisation (CSIRO), Canberra, Australian Capital Territory, Australia; 2 Business Methods Survey Division, Statistics Canada, Ottawa, Ontario, Canada; 3 McGregor GeoScience, Bedford, Nova Scotia, Canada; Instituto de Biologia, Brazil

## Abstract

The ability to quantitatively assess ecological health is of great interest to those tasked with monitoring and conserving ecosystems. For decades, biomonitoring research and policies have relied on multimetric health indices of various forms. Although indices are numbers, many are constructed based on qualitative procedures, thus limiting the quantitative rigor of the practical interpretations of such indices. The statistical modeling approach to construct the latent health factor index (LHFI) was recently developed. With ecological data that otherwise are used to construct conventional multimetric indices, the LHFI framework expresses such data in a rigorous quantitative model, integrating qualitative features of ecosystem health and preconceived ecological relationships among such features. This hierarchical modeling approach allows unified statistical inference of health for observed sites (along with prediction of health for partially observed sites, if desired) and of the relevance of ecological drivers, all accompanied by formal uncertainty statements from a single, integrated analysis. Thus far, the LHFI approach has been demonstrated and validated in a freshwater context. We adapt this approach to modeling estuarine health, and illustrate it on the previously unassessed system in Richibucto in New Brunswick, Canada, where active oyster farming is a potential stressor through its effects on sediment properties. Field data correspond to health metrics that constitute the popular AZTI marine biotic index and the infaunal trophic index, as well as abiotic predictors preconceived to influence biota. Our paper is the first to construct a scientifically sensible model that rigorously identifies the collective explanatory capacity of salinity, distance downstream, channel depth, and silt–clay content–all regarded a priori as qualitatively important abiotic drivers–towards site health in the Richibucto ecosystem. This suggests the potential effectiveness of the LHFI approach for assessing not only freshwater systems but aquatic ecosystems in general.

## Introduction

Assessment of the “health” of an ecosystem is often of great importance to those interested in the monitoring and conservation of ecosystems. Health is a complex concept often involving many diverse factors, and therefore is not straightforward to quantify. A popular method to estimate ecosystem health is through one or more multimetric indices, each of which is a scalar collapsed from several indicator variables of health, or metrics. Often, ecosystem health metrics are measures of faunal abundance and diversity. For aquatic ecosystems, these biotic metrics typically focus on benthic populations because they are useful indicators of underlying health conditions [1], [2]. For example, the AZTI marine biotic index (AMBI) [3] is a quantitative measure of health for an estuarine ecosystem based on the sample counts of categorized benthos. Its popularity is evident from its use across the globe, including Africa [4], Asia [5], Europe [6], North America [7], and South America [8].

AMBI and other common multimetric indices, e.g., infaunal trophic index (ITI) [9], estuarine biotic integrity index [10], benthic response index [11], benthic quality index [12], infaunal quality index [13], have the main appeal that they are conceptually simple and thus easily interpretable. They also contain a high amount of biological content from subject-matter scientists being involved at all stages of the design of the index. Yet, the construction and mathematical formulation of many such indices can involve a substantial amount of investigator-specific definitions that are qualitative in nature. Consequently, rigorous evaluation of index reliability and other quantitative aspects is difficult with conventional indices: for example, detecting relationships between health and environmental or impact-related covariates such as water depth or urbanization; and formally assessing the uncertainty in these estimates of health. Recent multistep approaches towards addressing such concerns (e.g., [11], [14]) do not address propagation of uncertainty from one step to another, thereby resulting in inference that is less reliable than that from an integrated statistical methodology. Chiu and Guttorp [8] proposed the SHIPSL approach, a statistically enhanced method to construct multimetric indices. Dobbie and Dail [16] compared SHIPSL with other stream health index approaches through a simulation study and showed SHIPSL to have the most favorable statistical properties. Nonetheless, SHIPSL and conventional multimetric approaches share unresolved issues such as being space- and/or time-specific, and the need for follow-up analyses to determine its relationship with nonfaunal (abiotic) variables in method evaluation or policy-making contexts.

Recently, Chiu et al. [17] devised the latent health factor index (LHFI), a novel statistical model-based ecological index aimed to retain the advantages of conventional multimetric indices while addressing some of their shortcomings. In [17], the LHFI modeling methodology was demonstrated and validated on freshwater ecosystems. Through M.W.'s master's studies [18], we adapted this approach to assess an estuarine ecosystem, utilizing the dataset collected by Lu et al. [19] in the previously unassessed Richibucto estuary in the Canadian province of New Brunswick. The LHFI approach involves a multilevel analysis of covariance generalized linear mixed-effects (regression) model (e.g., [20]), or ANOCOVA GLMM: instead of being treated as *measures* of health, metrics are regarded as *indicators* of underlying health conditions. Thus, metrics are regressed as response variables upon a latent health quantity (latent since it is not directly observable) which is site-specific, forming the main level of the regression; health in turn can be regressed upon available drivers/covariates, such as environmental (e.g., salinity, silt–clay content) and impact-related (e.g., urbanization) variables, forming the optional sublevel in the model hierarchy.

With data on metrics and covariates, latent health can be estimated as a scalar, so that interpretability is retained; the estimated quantity is the value of the index. Additionally, the effect of drivers on health can be evaluated in a single integrated statistical framework. Regressing abundance/richness metrics directly on drivers is common in the literature (e.g. [21], [22]). Yet, with latent health additionally sandwiched between metrics and drivers, the LHFI regression hierarchy naturally expresses the abstract notion of health as a quantitative parameter, thus integrating the formal quantification of health with attributing it to drivers. Importantly, statistical modeling is what directly produces the health index under the integrated LHFI framework, as opposed to being employed merely to select relevant metrics before index construction (e.g., in [10]) or to evaluate the resulting index (e.g., in [23]). Thus, the LHFI is much more rigorous than conventional indices, as its definition utilizes universal modeling practices for the definition of the index; its hierarchical modeling framework also allows comprehensive statistical inference without the need for sequential analyses through which the propagation of uncertainty is lost from one analysis to the next. As well, the approach provides a predictive framework under which interpolation of health for a new site can be carried out in a cost effective yet rigorous fashion. Specifically, once an appropriate LHFI model has been identified for a set of existing sites, prediction a posteriori can be accomplished simply with covariate values observed at this new site, thus bypassing the expensive benthic taxonomic laboratory procedures that are required to gather the metric data as required by conventional indices. These desirable properties are gained without sacrificing scientific integrity in the form of subject-matter expertise, which can be involved in the identification of biologically relevant metrics and covariates to form the LHFI. It is also straightforward to use the LHFI framework to handle data that have certain types of spatial and/or temporal features, thus resolving the space/time-specific issue of other indices.

Recently in [24], LHFI principles were integrated with formal point-referenced spatial modeling [25] to formulate the hierarchical relationship among four levels of quantities: (i) ordinal health metrics each on a five-point scale from “poor” to “excellent,” (ii) latent continuous quantities that determine the ordinal metrics, (iii) latent health, and (iv) geographical/environmental covariates. This formulation illustrates the type of unified statistical inference that can be drawn from such an LHFI-based approach for assessing biotic integrity of river basins in Colorado, USA. In contrast, directly modeling the quantitative health indicators based upon which ordinal metrics are defined [17] can avoid the loss of information due to mapping quantitative health metrics to a coarse ordinal scale. This was the approach for our preliminary models, but they had a major limitation: estuarine health was statistically attributable to separate subsets of ecologically important drivers, but when these subsets were integrated into a single LHFI model, all but one driver came out statistically significant. In the following sections, we first discuss our preliminary estuarine LHFI models and main findings (crux of M.W.'s studies [18]). We then proceed to build on these models by considering two possible extensions: (a) a nontrivial covariance structure, and (b) additional level(s) to the regression hierarchy based upon the known associations among various drivers.

## Methods

### Constructing Estuarine LHFIs

**Figure 1:**
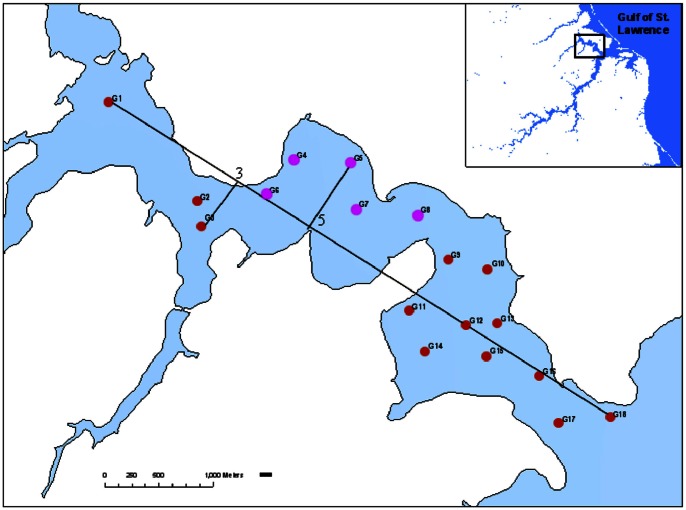
Map of Richibucto Estuary. The 18 monitored sites are shown in red/pink and labeled “G” followed by the site number. Red sites were sampled in September and pink ones, in October. Straight lines illustrate the method for calculating *distance downstream* (DD) for Sites 3 and 5.

Our data (Appendix S1) were collected by Lu et al. [19] in the Richibucto estuary at 18 sites (Figure 1) who used these data to investigate the relationship between soft-bottom macrobenthic communities and environmental variables. Macrofaunal data–88 species for the estuary–were recorded from 2–3 grab sample replicates per site collected between September (Sites 1–3 and 9–18) and October (Sites 4–8) in 2006. Many dominant species were polychaetes, oligochaetes, amphipods, gastropods, and bivalves (the top five dominant species for each site appear in Table 2 in [19]). Observed alongside benthic fauna were abiotic properties of the estuary (Table 1 in [20]). They included *depth*, the distance (m) from the water surface to the estuary bed at the location of the site from which grab samples were obtained; *water temperature* (°C) and *salinity* (parts per thousand), both measured from a single in situ water sample obtained at the site; SC, the *fraction of silt–clay* (grains of size 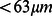); *median grain size* of sediment; *sorting* (a unitless measure of variability of grain size); and *organic content* (%). The latter four variables were recorded by extracting two subsamples from each grab replicate, then pooling all subsamples for sediment assay.

**Table 1:** Metrics based on the definition of AMBI, used to construct LHFIs for the Richibucto estuary.

Metric Number	AMBI Abundance^a^ Metric	Preconceived Association with Health
1	species (including specialist carnivores and some deposit-feeding tubicolous polychaetes) verysensitive to organic enrichment and present under unpolluted conditions	+
2	species (including suspension feeders, less selective carnivores and scavengers) indifferent toenrichment, always present in low densities with nonsignificant variations with time	±^b^
3	species tolerant to excess organic matter enrichment (including surface deposit-feedingspecies, e.g., tubicolous spionids)	−
4	second-order opportunistic species; mainly small-sized polychaetes: subsurfacedeposit-feeders, e.g., cirratulids	−
5	first-order opportunistic species: deposit-feeders, which proliferate in reduced sediments	−

a Organisms with the specified characteristics, given all benthic organisms in the grab sample.

b Neither clearly positive nor clearly negative.

**Table 2:** Metrics based on the definition of ITI, used to construct preliminary LHFIs for the Richibucto estuary.

Metric Number	ITI Abundance Metric*	Preconceived Association with Health
1	suspension feeders: feed on detritus from the water column and usually lack sediment grainsin their stomach contents	+
2	interface/surface detrital feeders: obtain the same types of food as suspension feeders but usuallyfrom the upper 0.5 cm of the sediment	+
3	deposit feeders: invertebrates (including carnivores); generally feed from the top few cm of thesediment and feed on encrusted mineral aggregates, deposit particles or biological remains	±
4	specialized environment feeders: mobile burrowers that feed on deposited organic material;all adapted to live in highly anaerobic sediment	−

* As described in [35].

Sites 2, 4–7, and 14 in the estuary were closest to active oyster farms [19]. Oyster farming activity is perceived to impact site health through its direct influence on sediment properties, although different biotic indicators were reported to show different types of association with proximity to oyster farms [19]. For example, relative to all 18 sites, macrobenthic faunal abundance was moderate for Sites 4–7 and 14 but high for Site 2, while Shannon's diversity [26], [27] is relatively even among all sites aside from a slight upward trend with increasing distance from the upper channel instead of from an oyster farm. Even when Lu et al. [19] considered the abundance of various dominant species as a suite of separate indicators, they saw no obvious association between these latter indicators and oyster farm location. Shannon's index has limitations including ambiguity in its interpretation [26], [28]; the same is true for other nonmodel-based indicators such as ones based on single species. This motivated us to build LHFI models for Richibucto based on indicator metrics (Tables 1–2) used to construct the AMBI and ITI. Specifically, AMBI and ITI metrics are popular estuarine ecosystem health indices, being better tailored for estuaries than the generic indicators of abundance, richness, and diversity; and they are more comprehensive than indicators based on single species. However, biotic health indicators alone do not explicitly reveal the collective impact on overall health from abiotic variables: benthic fauna in Richibucto are believed to be related to organic enrichment (plausibly affected by oyster farming activity), freshwater input (salinity gradient), variability of sediment particle size, water temperature, and topography (channel and water depth), as well as their interactions [19]. To this end, we considered two sets of preliminary LHFI models. The first included only metrics from AMBI (denoted by LHFI-A), and the second, metrics from both AMBI and ITI (denoted by LHFI-A-I).

### Identifying Drivers of Estuary Health

For each of LHFI-A and LHFI-A-I, we investigated which and how covariates might influence site health as reflected by biotic metrics. As discussed in [17], a thorough understanding of the relationship between covariates and health is key to rigorous yet cost effective interpolation of site health. Indeed, interpolated biotic conditions would be unreliable when the LHFI model includes weakly predictive abiotic covariates, such as an environmental gradient that exhibits little change across the study area. On the other hand, an LHFI model with good predictive power could prove to be an enormous asset to biologists and policy makers for biomonitoring purposes.

To this end, we implemented preliminary LHFI-A and LHFI-A-I models with different combinations of the covariates listed in the previous subsection, as well as two additional candidates: *month* (September or October) and DD, the *distance downstream* (km). DD is measured by extending a straight line from the western-most site (Site 1) to the eastern-most site (Site 18), then defining DD for any site as the distance between the site's perpendicular projection onto the straight line and Site 1 (Figure 1, Table 3). An alternative covariate to DD would be two-dimensional spatial coordinates of sites. Though, as shown in Figure 1, the study sites roughly align diagonally across a small geographical domain of approximately (4km)×(23km). Thus, we expect little loss of information through collapsing the two-dimensional coordinates into DD. In fact, this single spatial covariate can avoid collinearity between the spatial dimensions.

**Table 3:** Distance downstream (km) for Richibucto sites.

Site	1	2	3	4	5	6	7	8	9
Distance	0	1.164	1.298	1.731	2.179	1.686	2.463	2.970	3.433
Site	10	11	12	13	14	15	16	17	18
Distance	3.790	3.358	3.880	4.119	3.642	4.179	4.701	5.060	5.448

These preliminary models were considered in a Bayesian statistical framework, as follows. For LHFI-A, AMBI metrics are abundances of five disjoint taxonomic groups. We denote the metrics by 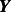. Due to the difference in the preconceived direction of their association with health (Table 1), we split the metrics into two groups: 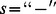 for Metrics 3–5 (negatively related to health), and 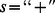 for the remaining metrics. In the LHFI model, each member of Group 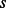 is modeled as a multinomial random variable. The link function for the GLMM is a generalized logit for 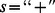, and an inverted generalized logit for 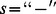. Thus, large metric values for 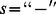 and “+” reflect, respectively, poor health and otherwise. More precisely, let 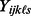 denote the value of the 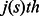 metric (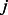 nested in the 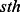 group) for the 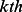 replicate grab sample at the 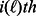 site (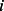 nested in the 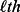 month for 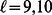). Let 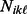 be the total number of benthic organisms in the 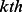 replicate sample at the 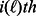 site, and 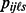 be the unknown probability that a random organism from the 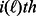 site belongs to the 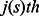 taxonomic group. Thus, we have multinomial distributions.
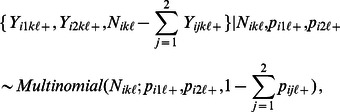
(1)

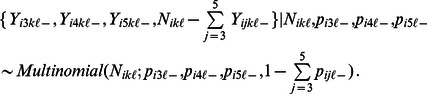
(2)


Next, let 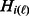 denote the latent health of the 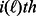 site; and 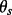 and 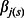 respectively denote the metric group effect and individual metric effect (both unknown) in the regression model. Then, the linear predictor in the LHFI framework is.
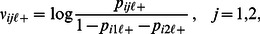
(3)

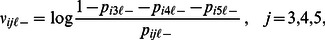
(4)

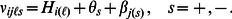
(5)


For Equation 5, we model 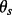 as a fixed effect and take 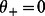 (as is customary when considering one of the categories as baseline) to ensure model identifiability, and we model site health and metric effects as random. Note that there is overlap and thus dependency between the two multinomials of Equations 1 and 2. This dependency is crudely accounted for by 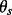; similarly, metric effects 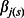 crudely account for the dependency among 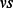 within group 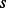. Thus, for these preliminary models, we assumed independent mean-zero Gaussian 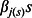, while allowing for unequal variances across 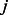.

Finally, the latent regression of 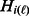 is.
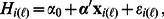
(6)

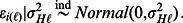
(7)where 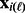 is the vector of a given combination of the aforementioned covariates, 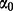 and 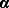 are the unknown coefficients of the corresponding latent regression, and 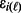 is the normally distributed regression error with unknown variance 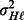 that may vary over months. In practice, covariate transformation might be necessary to satisfy the linearity of Equation 6. Covariates (possibly transformed) are then centered to reduce dependence among the 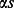. For a given covariate that is not an interaction, centered data are produced by subtracting from the raw covariate data a constant that is (approximately) equal to the observed covariate mean (averaged over 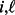). The “centered interaction” between two covariates is taken to be the product of two centered covariates. For example, the centered 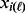 terms in Equation 6 corresponding to log-SC, log-depth, and their interaction would be computed, respectively, as 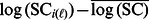, 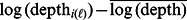, and 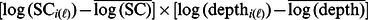.

The formulation of Equations 6 and 7 does not depend on the biotic metrics. Thus, for LHFI-A-I, we include additional ITI elements that correspond to Equations 1–[Disp-formula pone.0065697.e025] [Disp-formula pone.0065697.e030] [Disp-formula pone.0065697.e031] 5. Briefly, the combined framework is as follows. The ITI counterparts of Equations 1–[Disp-formula pone.0065697.e025] [Disp-formula pone.0065697.e030] [Disp-formula pone.0065697.e031] 5 are based on partitioning ITI metrics into “+” and 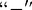 groups according to Table 2. For combining AMBI and ITI metric effects, we replace 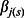 with 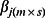 where 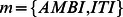. This reformulated 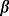 is decomposed as the sum of three components: a fixed effect due to 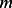, a fixed effect due to the interaction between 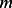 and 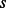, and a random effect due to 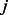 nested inside 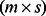. For our preliminary models, these latter random effects took the role of 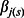
s from the AMBI-only case. Appendix S2 presents more details on the LHFI-A-I framework.

We implemented the above modeling framework using Markov chain Monte Carlo (MCMC) techniques. Several covariates and interactions exhibited a statistically significant relationship with health (Bayesian credible intervals that excluded the regression coefficient value of 0 had a credible level that was reasonably high, e.g., 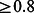). For LHFI-A, two best-fitting models were identified among those investigated: one with abiotic covariates log-SC, log-depth, their interaction, and salinity; and another with the single covariate DD. The remaining covariates, including month, were found to be insignificant or confounded with others. We observed little evidence that 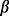 variances were unequal, and thus assumed constant variance 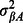 for AMBI metric effects when formulating the integrated LHFI-A-I model (see Appendix S2). Under this formulation, there were three best LHFI-A-I models: two corresponded to the same sets of significant covariates as those for LHFI-A, and another model with covariates log-depth, log-SC, and their interaction. For both LHFI-A and LHFI-A-I, we observed some evidence that 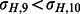.

However, our attempts to include covariates from various best-fitting models together in a single LHFI-A or LHFI-A-I model were unsatisfactory. In such combined models, DD remained highly significant, while all other covariates and their interactions were no longer significant at a reasonable credible level. Indeed, salinity and DD are highly correlated (Table 4), and the two cannot be simultaneously significant due to collinearity. However, no strong correlation exists among log-depth, log-SC, and DD (Table 4), and so why did DD eclipse all others in a combined model, despite nonDD covariates being significant when DD was absent? As well, while relationships between health and covariates were quite strong for the LHFI-A models, they were less clear for LHFI-A-I models (significance at credible levels 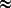 60–85% in the best-fitting LHFI-A-I models, as opposed to 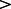 90% for LHFI-A). This indicated that the extra data from ITI metrics weakened the overall relationship between health and covariates. One possible explanation for this phenomenon is that the LHFI construct was appropriate for describing health using AMBI metrics and the available covariates, but ITI metrics have weak ecological relevance to Richibucto. This is plausible from a qualitative perspective, in light of our prior beliefs about health drivers as stated above. One remedy is to determine additional covariates that can be more appropriately paired with ITI metrics, then model these alongside the original covariates. However, this would require further field activities, and is beyond the scope of our current paper. Thus, for the remainder of this paper, we focus on addressing the domination of DD for LHFI-A only.

**Table 4:** Sample correlation coefficients among covariates for latent health of Richibucto sites.

	DD	salinity	log-depth	log-SC
DD	1	0.88	0.16	−0.47
**salinity**		1	0.23	−0.33
**log-depth**			1	−0.41
**log-SC**				1

Indeed, the above preliminary models might be improved upon. Specifically, distance likely contained much less measurement error than the other covariates, it being easier to measure with precision than the environmental covariates which are intrinsically more variable in nature. With a simplistic LHFI model, the effect from distance on health could therefore manifest itself more clearly than effects from other covariates even if all of them were equally important in a qualitative sense. Given our prior beliefs about AMBI metrics being more relevant to Richibucto, and the fact that environmental covariates contained ecological information that distance did not, a more sophisticated LHFI modeling framework may be helpful in providing a common thread through health, DD and the other ecologically relevant covariates.

To this end, we proceed to determine if either of the following helps to clarify the nature of the relationship among latent health and the available covariates: (1) Introduce a covariance structure for the metric effects, instead of independence which was assumed for the preliminary models to reduce computational burden; (2) introduce additional level(s) to the regression hierarchy based upon the known associations between the available covariates. These steps pertain to different parts of the LHFI model, and thus we treat each as a stand-alone investigation. Note that even if ITI metrics had shown to be highly relevant to estuary health in Richibucto, introducing extra model complexity to LHFI-A-I models can be impractical for proper inference via MCMC. This is because AMBI and ITI metrics are dependent according to their definitions, so that extra model parameters are required to account for this. Even when such dependence is only informally accounted for by the fixed-effects terms in the LHFI-A-I formulation in Appendix S2, one can see the substantial extra complexity that is required.

### Extending LHFI-A via a Nontrivial Covariance Structure for Metric Effects

Recall that our preliminary LHFI-A models provided little evidence that 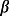 variances were unequal; subsequently we took 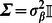, where 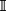 is the identity matrix. To generalize 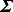, we now replace independence of metric effects by.
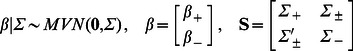
(8)where 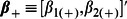, 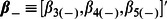, 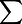 is the unknown covariance matrix for 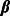, and “MVN” denotes the multivariate normal distribution. Thus, 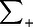 (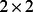), 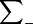 (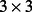), and 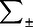 (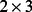) denote the covariance matrices for metric groups positively and negatively related to health, and their cross-covariance matrix, respectively. (Note that any 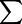 structure in Equation 8 necessarily differs from the posterior covariance structure for 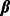.) With a small dataset from 18 Richibucto sites each with only 2 to 3 replicate grab samples, a practical concern is that a general 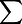 may be only weakly identifiable depending on the complexity of the covariance structure (see [15] for a discussion on lack of identifiability in Bayesian inference). This issue was encountered in [9] when a fully unstructured 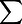 was assumed for a freshwater benthic dataset that also involved 18 sites with 3 replicates per site, but with nine metrics altogether. To avoid weak identifiability, one could consider a structured 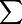, as discussed in Appendix S3, to reduce the number of unknown parameters; a special case is the block diagonal 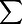 with 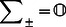 in Equation 8. To further reduce inferential burden, we additionally assume 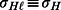 to be constant over months. This assumption effectively removes the index 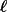 from the entire model, and may be justified by the possibility that the preliminary evidence for 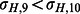 was related to the lopsided abundance of data from September (13 sites) compared to October (5 sites).

Overall, statistical inference is focused on ecologically pertinent parameters, namely, 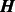, 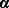, and 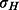; 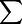 and other parameters are regarded as nuisance. For Bayesian inference, we use relatively diffuse distributions as priors for 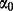, elements of 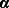, 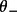 (univariate Gaussians, each with mean 0 and variance 100), and 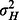 (inverse-Gamma with unit shape and scale). Diffuseness of priors reflects the fact that in the absence of data, we have no clear perception of the properties of the corresponding unknown quantities. In general, diffuseness reduces the need for justification of prior distributional assumptions. To complete the Bayesian modeling hierarchy, we must specify the priors for 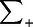 and 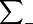. The most general form is for each to be unstructured, and thus we take.
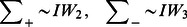
(9)where 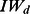 is the inverse of a 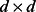 random Wishart matrix with 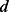 degrees of freedom and scale matrix equal to the identity, which is a relatively diffuse prior for a 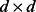 unstructured covariance matrix. Then, one can take advantage of existing MCMC software such as OpenBUGS [21] for straightforward implementation of the LHFI model, although in our experience, nontrivial hierarchical centering is essential to improve MCMC mixing [9], [34]. Altogether, the extended model comprises Equations 1–[Disp-formula pone.0065697.e025] [Disp-formula pone.0065697.e030] [Disp-formula pone.0065697.e031] [Disp-formula pone.0065697.e032] [Disp-formula pone.0065697.e042] [Disp-formula pone.0065697.e043] [Disp-formula pone.0065697.e076] 9 with 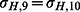.

### Extending LHFI-A via an Extra Level in the Latent Regression

**Figure 2:**
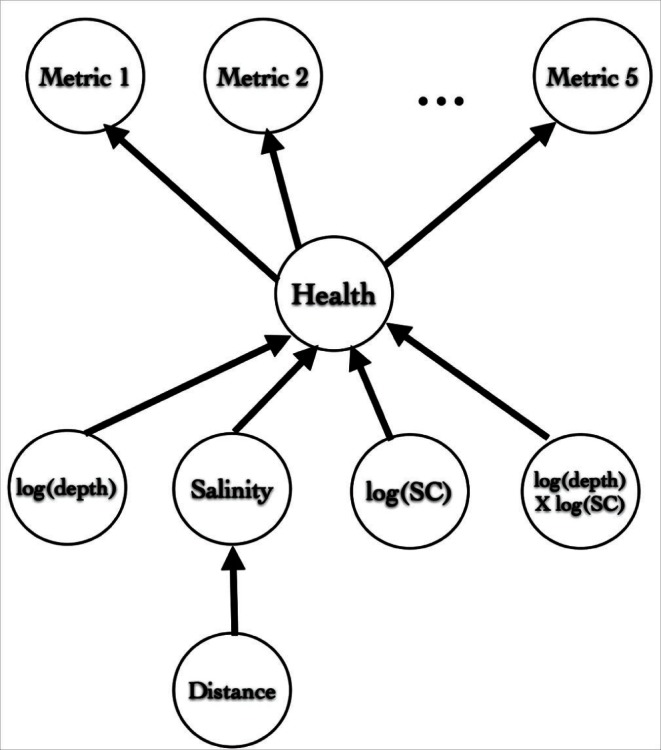
Regressing salinity on distance downstream as an additional level in the hierarchical latent health model. Distance downstream (DD) is the driver of salinity. Salinity and other covariates are complementary drivers of latent health, which is a driver of AMBI metrics.



Extra model complexity can be introduced also through an additional level in the regression of latent health on covariates. Specifically, although the strong correlation between salinity and DD reflects ecological reasoning for coastal sea waters entering an estuary, it is the only clear empirical relationship detected among the available covariates. Therefore, instead of considering salinity and DD to be complementary covariates, we now take salinity as a response of DD, and in turn, latent health as a response of salinity and the remaining covariates identified in the preliminary analyses as statistically significant (Figure 2). Then, the LHFI model comprises Equations 1–[Disp-formula pone.0065697.e025] [Disp-formula pone.0065697.e030] [Disp-formula pone.0065697.e031] [Disp-formula pone.0065697.e032] [Disp-formula pone.0065697.e042] [Disp-formula pone.0065697.e043] 8 with 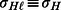 (see subsection above), plus.

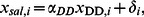
(10)

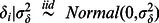
(11)where 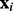 in Equation 6 denotes the vector of centered covariates for site 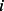 including salinity 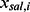 (and possibly other covariates) but excluding DD 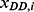. Hence, Equations 6 and 10 can be collapsed into
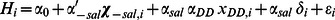
(12)where 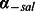 is 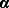 with 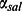 (and 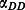) removed, and similarly for 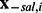. Thus, Equation 12 regards salinity as an implicit covariate, so that when latent health is explicitly regressed on 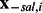 and DD, the implicit covariate decomposes the total error variation into 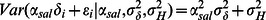. Hence, a smaller ratio 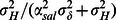 reflects a higher contribution from the implicit covariate towards explaining the total error variation of the latent health regression.

We again employ an inverse-Gamma prior with unit shape and scale for 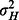, and also for 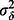. Univariate Normal(0, 100) priors are employed for 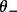, 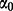, and elements of 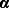, with one exception: 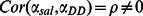 is additionally considered, where 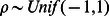 a priori.

## Results

Results of our preliminary LHFI-A and LHFI-A-I models already appear under **Identifying Drivers of Estuary Health**. Below, we report the results of our extended LHFI-A models.

### Health Inference as a Whole is Robust to Metric Covariance Structure

**Figure 3:**
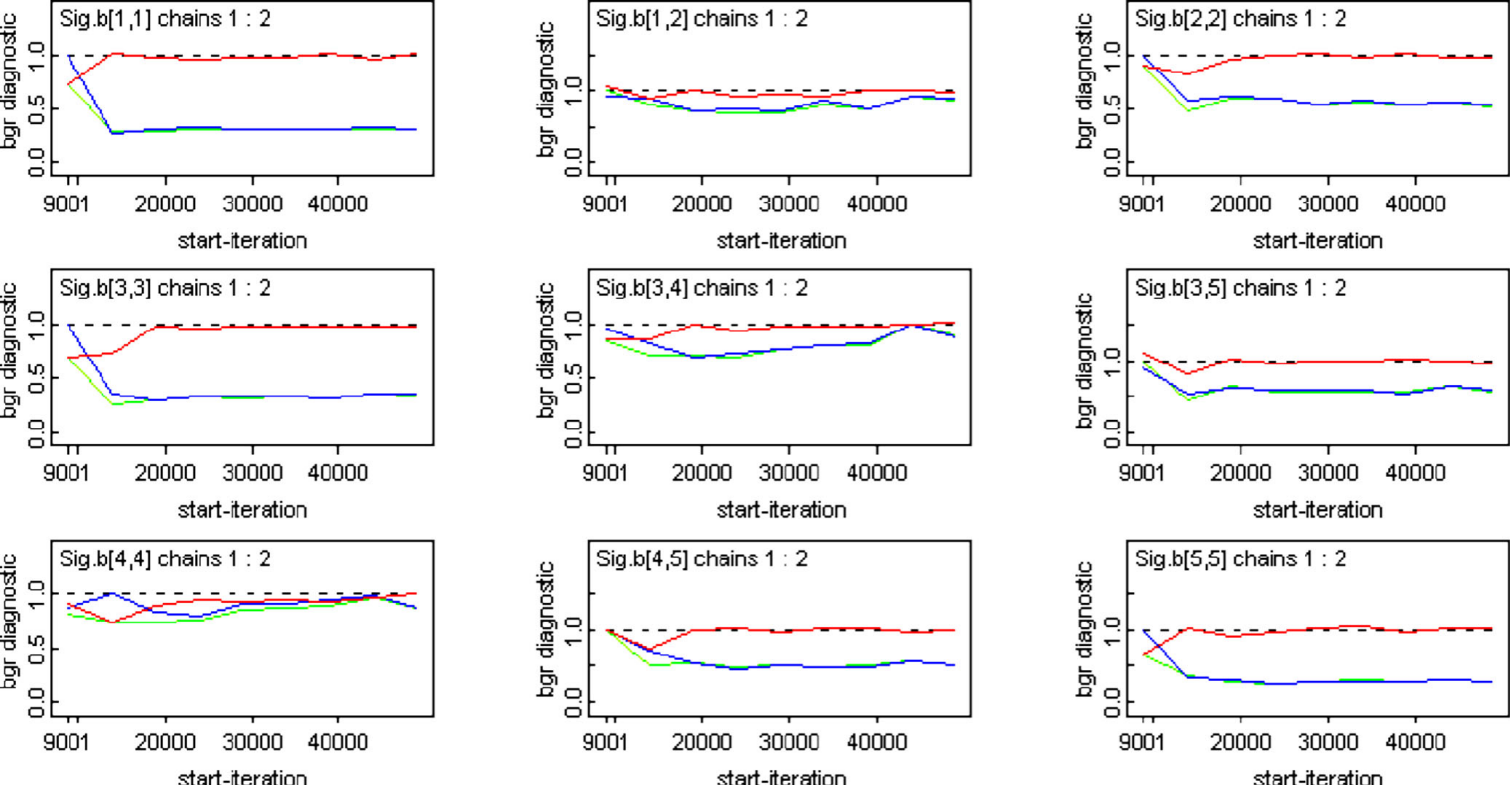
Brooks-Gelman-Rubin (BGR) diagnostics for the block diagonal ***Σ*** **.** BGR plots are presented for the MCMC samples of each nonzero matrix element 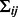 (axis label Sig.b[i,j] in plot), i.e., the 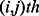 element of 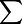. Convergence is suggested by a red curve approaching 1, together with green and blue curves approaching the same constant [34].

**Figure 4:**
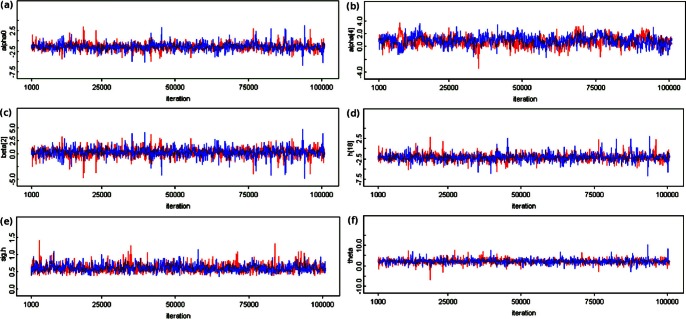
Trace plots of two independent MCMC chains. The two chains for selected parameters from fitting Equations 3–[Disp-formula pone.0065697.e031] [Disp-formula pone.0065697.e032] [Disp-formula pone.0065697.e042] [Disp-formula pone.0065697.e043] [Disp-formula pone.0065697.e076] 9 (assuming 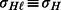) are shown in red and blue, both thinned by 100 iterations. (a) Intercept 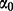. (b) Regression coefficient of the (centered) interaction 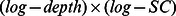. (c) Random effect 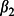. (d) Latent health 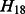. (e) Standard deviation 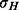. (f) Fixed effect 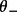. Trace plots for all other 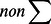 model parameters show similar patterns that suggest convergence after a burn-in of merely 1,000.

We considered the LHFI-A model with covariates log-depth, log-SC, their interaction, and DD, which were identified from our preliminary models as the most statistically relevant covariates while assuming 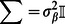. Our Bayesian estimates for parameters of main interest and corresponding credible intervals then were compared to their preliminary counterparts. The result was that increasing complexity of the LHFI-A model through a nontrivial 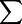 did not lead to a noticeable difference in the significance of the covariates or the posterior mean of 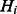. In general, extra model complexity could lead to overfitting, which in turn leads to weaker model inference. In light of the concern over weak identifiability as explained above, we would expect weaker model inference to manifest itself in the form of MCMC mixing difficulties for 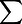 despite having employed hierarchical centering. However, this was not the case for our analysis, as two independently generated MCMC chains mixed readily after a manageable burn-in. In particular, although parameters of 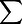 could require a burn-in of up to approximately 20,000 iterations (Figure 3), all other model parameters each required a burn-in of only 1,000 or less (Figure 4). (For a given model, inference for model parameters as a whole was always based on the longest burn-in required.) Instead, weaker inference was apparent only in the form of slightly larger posterior dispersions for 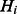 and certain nuisance parameters when compared to the case of 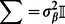. Therefore, neither the relative health rankings among sites (even accounting for wider credible intervals) nor the identification of significant health drivers was affected by assuming a more complex structure for 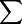. Our investigation here suggests that the inference for latent health 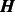 associated with the Richibucto system is reasonably robust to the prior covariance structure in Equation 8 for metric effects 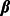. Consequently, for model parsimony, we regard 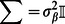 (as we had originally assumed) to be adequate for these Richibucto data.

### Simultaneously Significant Covariates in Two-Level Health Regression

**Figure 5:**
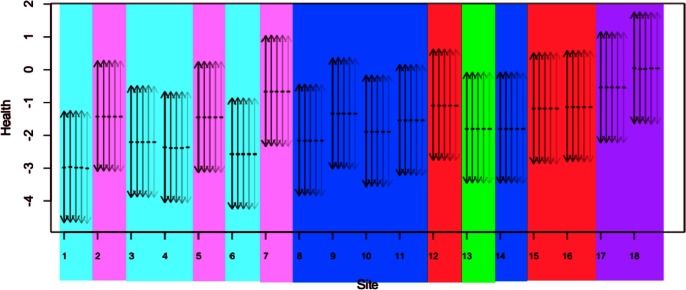
LHFI scores and corresponding 95% CIs of site health. Health scores (posterior means marked by “–”) and CIs (arrows from dark to light) are based respectively on Models (1), (2), (3), (4), and (5) of Table 5. Lu et al. [20] partition Richibucto sites into six groups according to their benthic community composition: red (lower channel: Sites 12, 15, 16), pink (upper channel: Sites 2, 5, 7), violet (estuarine mouth: Sites 17, 18), blue (lower shallow: Sites 8–11, 14), turquoise (upper shallow: Sites 1, 3, 4, 6), and green (other: Site 13).

**Figure 6:**
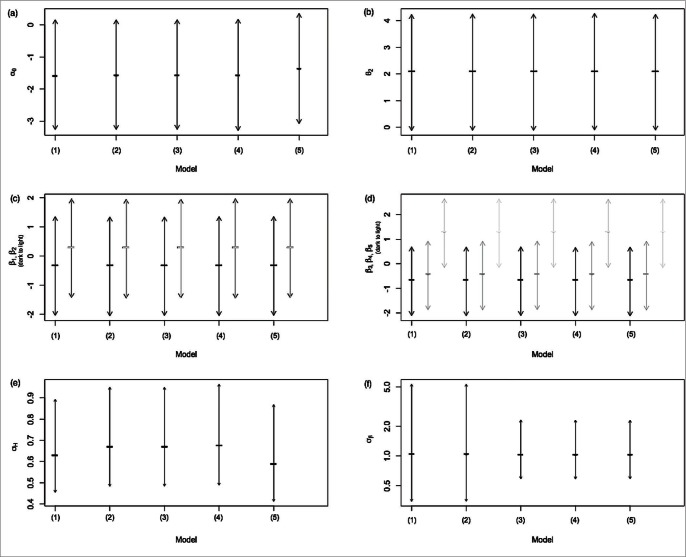
Posterior means and 95% CIs for dispersion parameters and nuisance regression coefficients. Posterior means are marked by “–” inside the CIs. (a) Intercept 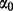. (b) Fixed effect 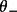 (axis label 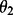). (c) Elements of 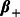. (d) Elements of 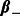. (e) Standard deviation 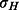. (f) Standard deviation 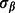 (plotted on log-scale).

Table 5 and Figures 5 and 6 present inference summaries assuming 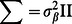 and 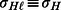 for various LHFI-A models. Models (3)–(5) each comprises two levels of covariates (Equations 1–[Disp-formula pone.0065697.e025] [Disp-formula pone.0065697.e030] [Disp-formula pone.0065697.e031] [Disp-formula pone.0065697.e032] [Disp-formula pone.0065697.e042] [Disp-formula pone.0065697.e043] 8 and 10–11). Models (1) and (2), provided for comparison, each comprises a single level of covariates (Equations 1–[Disp-formula pone.0065697.e025] [Disp-formula pone.0065697.e030] [Disp-formula pone.0065697.e031] [Disp-formula pone.0065697.e032] [Disp-formula pone.0065697.e042] [Disp-formula pone.0065697.e043] 8 only). Posterior means for latent health along with their 95% posterior credible intervals (CIs) appear in Figure 5; those for 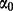, 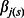, 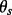, 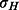, and 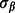 appear in Figure 6. Note that in addition to 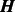, 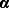, and 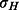, here 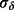 from the extra level in Models (3)–(5) is also a parameter of ecological interest.

**Table 5:** Selected summary statistics of posterior draws.

						95% CI	
Model	Description	DIC	Parameter	Mean	Median	2.5%	97.5%
(1)	*DD* only	4380					
			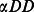	0.37	0.37	**0.16**	**0.58**
			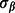	1.48	1.03	0.35	5.32
			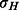	0.63	0.61	0.45	0.89
(2)	*sal* only	4379					
			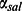	0.39	0.39	**0.13**	**0.64**
			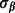	1.48	1.02	0.35	5.32
			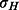	0.67	0.66	0.48	0.95
(3)	*sal*-on-*DD* only; 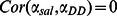	4417					
			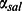	0.39	0.39	**0.13**	**0.64**
			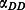	0.77	0.77	**0.54**	**1.00**
			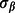	1.12	1.01	0.59	2.31
			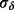	0.70	0.68	0.51	0.98
			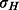	0.67	0.65	0.48	0.95
			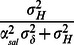	0.54	0.55	0.31	0.76
(4)	*sal*-on-*DD* only; 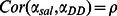	4419					
			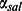	0.49	0.49	**0.28**	**0.71**
			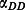	0.59	0.59	**0.35**	**0.79**
			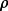	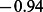	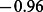	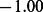	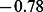
			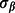	1.12	1.01	0.59	2.30
			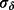	0.74	0.72	0.53	1.07
			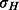	0.68	0.66	0.49	0.96
			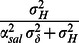	0.59	0.59	0.37	0.79
(5)	log-*depth*, log-*SC*, 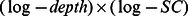 , and *sal*-on-*DD*; 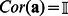	4417					
			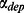	0.16	0.16	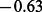	0.94
			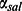	0.42	0.42	**0.17**	**0.67**
			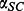	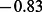	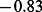	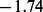	0.08
			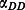	0.77	0.77	**0.54**	**1.00**
			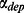 × 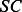	1.88	1.88	**0.28**	**3.49**
			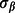	1.12	1.01	0.59	2.29
			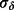	0.70	0.68	0.51	0.98
			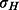	0.59	0.57	0.41	0.87
			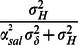	0.63	0.63	0.37	0.87

Boldfaced CI limits indicate that the slope or correlation parameter differs from 0 with at least 95% credibility.

It is evident from the 95% CIs in Table 5 that, when DD is considered a driver of salinity, the two are simultaneously relevant to explaining latent health. In fact, 0 is excluded from the 99% CI (not shown) for both 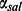 and 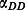 in each of Models (3)–(5), suggesting very high credibility for the covariates in a two-level structure. The CIs from Model (5) suggest that the interaction 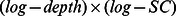 is an additional credible driver of latent health, complementing the explanatory capacity of salinity-on-DD. This is the first time that a scientifically sensible model has been successfully constructed to rigorously identify the collective explanatory capacity of salinity, DD, depth, and SC–all regarded a priori as qualitatively important–towards site health in the Richibucto ecosystem.

For Models (3)–(5), the posterior mean for the ratio 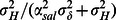 ranges respectively from around 0.55 to 0.65; corresponding 95% CIs span from 0.3+ to 0.8+. These moderately sized figures suggest that the salinity-on-DD structure is desirable for the model hierarchy, decomposing the total latent regression error variance into nontrivial components. Among Models (3)–(5), the former exhibits a smaller variance ratio, but not by much. Thus, despite the high credibility of the correlation between 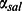 and 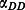 in Model (4) and of the influence on health from (the interaction between) depth and SC in Model (5), the least complex Model (3) provides slightly clearer evidence for the explanatory capacity of the two-level structure. In terms of the model's predictive power, the least and most complex among the three models share the same deviance information criterion (DIC) [30] which is slightly smaller (better) than that of Model (4). This predictive power corresponds to observed AMBI metrics (not latent health) being predicted by the model. To assess the model's predictive ability for latent health, one could conduct a simulation study in which unobservable 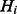 values are generated then estimated, although such an approach for hierarchical models has its shortcomings [22] or requires intensive computations [13] that may be impractical.

Instead, we compare CIs for 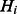 among models; in Figure 5, they appear nearly identical across all Models (1)–(5), i.e., the inference for health is essentially equally credible across various models. Within models, the relative ranking of sites according to their LHFI scores and associated CIs do not coincide with the clustering by Lu et. al [20], which was based on similarity in benthic community composition, and subsequently identified to be highly correlated with site location. Our results indicate that the LHFI approach does not merely represent community composition or site location; instead, it rigorously and comprehensively models biotic indicators, abiotic drivers, the abstract notion of health, and the relationship among them. Note that the health CIs from the 18 sites mutually overlap, suggesting that the small dataset does not allow us to distinguish sites according to their health at a 95% credible level; this was also the case for our preliminary models, all with single-level covariates. Despite (i) suboptimal distinguishability and (ii) weaker predictive power for AMBI metrics compared to the single-covariate Models (1)–(2), our two-level-covariate Models (3)–(5) clearly resolved the earlier counterintuitive phenomenon of qualitatively important covariates not being simultaneously significant. Indeed, (i) is an improvement over conventional methods in quantitative rigor due to the integrated manner from which our uncertainty estimates are obtained. Moreover, (ii) is of secondary concern when the response of key interest is 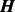 instead of the metrics 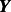. Aside from nearly identical latent health CIs across models, Figure 6 indicates that the five models perform equally well with respect to the uncertainty (width of CIs) of various dispersion parameters and nuisance regression coefficients, but with one exception: two-level-covariate models clearly yield less uncertainty for the inference of 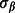 (Figure 6 (f)). As this parameter directly contributes to the uncertainty of the linear predictor 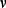, Figure 6 (f) indicates that the two levels can lead to more reliable prediction inference for faunal composition.

## Discussion

Unlike conventional multimetric health indices, the integrated LHFI approach employs hierarchical generalized linear mixed modeling to yield health scores, assess the influence of health drivers, and provide their associated uncertainty, all in a single, unified analysis for a given model. LHFI models can be tailored to different types of aquatic ecosystems through health metrics and environmental covariates that are specific to these systems. For example, while salinity can appear as a driver in an estuarine LHFI model, it would not be meaningful in a freshwater LHFI model (e.g., [17]) because of the lack of a salinity gradient in freshwater ecosystems.

For the Richibucto estuary, we constructed preliminary LHFI-A (with AMBI metrics only) and LHFI-A-I (with both AMBI and ITI metrics) models involving single-level covariates and independently distributed metric effects. A key goal of the preliminary models was to understand how biotic health indicators (AMBI and ITI metrics) might be driven by observed abiotic covariates, and in what combination of these covariates (main effects and interactions). However, our preliminary models lacked the important ability to rigorously identify relationships between health and drivers that are deemed ecologically important for the Richibucto system. In particular, if distance downstream were ignored, a combination of channel depth, salinity, and silt–clay content demonstrated high significance; distance downstream alone was significant when considered alongside other covariates. Subsequently, we considered two ways to explore this ecologically counterintuitive phenomenon: (a) to introduce a covariance structure on the random metric effects, and (b) to introduce additional levels of regression given preconceived relationships among the covariates. We implemented both (a) and (b) with AMBI biotic metrics only, but the approach would be applicable in principle to combining AMBI and ITI biotic metrics. Though, with merely 18 sites in Richibucto, our preliminary LHFI-A-I models suggested that ITI metrics potentially weakened any signal in the health-covariate relationship.

Based on our extended LHFI-A models, we have found (a) to be inconsequential to either the inference of latent health among Richibucto sites, or the lack of simultaneous statistical relevance of qualitatively important abiotic drivers of Richibucto health. On the other hand, (b) helped to rigorously express biological insight about these drivers: an additional level of covariates based upon the preconceived relationship between salinity and distance downstream has allowed the model to identify the simultaneous significance of distance and those abiotic covariates that our preliminary models had shown to be significant only when distance was excluded. Moreover, we have shown that model inference is more reliable overall when compared to single-level-covariate models. Thus, our two-level-covariate modeling framework more comprehensively exploits the ecological relationship among health, biotic metrics, and abiotic covariates, and it yields less uncertainty in model inference.

We implemented three variants of the two-level structure: (i) salinity-on-distance alone, with a priori independent regression coefficients and metric effects, (ii) same as (i) but assuming bivariate regression coefficients, and (iii) same as (i) but including channel depth and silt–clay content (both on the log scale), as well as their interaction. By decomposing the total latent regression error variance, the two-level structure successfully teased apart the explanatory contribution of salinity and distance, two highly collinear abiotic covariates. Overall, (i)–(iii) were almost equal in statistical performance, with slightly better predictive power of biotic metrics by (i) and (iii). Finally, while (i) corresponds to marginally stronger evidence for the two-level structure between salinity and distance to influence site health, (iii) confirms the simultaneous predictive power of all four ecologically relevant abiotic attributes. Our research demonstrates that LHFI modeling is flexible and can be an effective tool for assessing estuarine ecosystem health.

A technical note is that the LHFI framework is built on the fundamental principles of analysis-of-covariance, so that one can only interpret 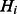 values in a relative sense. However, Chiu et al. [17] explain that including in the study any site that is qualitatively preidentified as very healthy or very unhealthy would facilitate the interpretation of the magnitude of 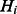 for an individual 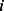. This is slightly different from the approach in [33] which includes sites that span the gradient spectrum of individual covariates. In general, any biologically relevant covariate that exhibits a substantial gradient across the domain of study should be a candidate for incorporation into the LHFI framework. On the other hand, a balance between model complexity and model parsimony is important to achieve reliable inference. For this reason, formal spatial models (point-reference or spatial random-effects models) were not considered for the small Richibucto dataset. Work is in progress at the Commonwealth Scientific and Industrial Research Organisation (CSIRO) to integrate spatial modeling into the LHFI framework for large spatial stream network datasets from eastern Australia. Like this paper, the work at CSIRO directly models the quantitative biotic metrics instead of their ordinal counterparts such as in [24].

Collecting and processing biotic data can be much more costly than abiotic data for the use in quantitative assessment of ecosystem health. Our research suggests that for the LHFI approach to rigorously distinguish sites according to AMBI and/or ITI metrics as indicators of estuary health, (1) more than 18 sites and/or (2) measurements made on abiotic covariates with higher precision may be needed. Though, implementation of (1) alone may suffice if abiotic data are recorded at additional sites even without collecting and processing faunal samples. This is because in the case of Richibucto, our approach led to an LHFI-A model (case (iii) above) which identified various abiotic predictors that are ecologically sensible and statistically relevant. Thus, one can (a) design health restoration experiments that focus on perturbing the identified abiotic predictors only, and (b) expect posterior interpolation of biotic conditions to be reliable, given these predictors. In general, the unified LHFI approach facilitates formal statistical interpolation of biotic conditions at sites for which abiotic information is observed. Thus, this methodology addresses key operational considerations in ecosystem management: guidelines for intervention of abiotic attributes and cost effectiveness of health inference without substantial tradeoff for statistical power. In light of its practical and statistical advantages, the LHFI methodology could be an invaluable asset if adopted for biomonitoring protocols by conservation biologists and environmental resource managers.

## Supporting Information

Appendix S1 **Richibucto data collected and studied by Lu et al.** [**19**] **.**
(PDF)

Appendix S2 **LHFI-A-I: integrating AMBI and ITI metrics.**
(PDF)

Appendix S3 **Some covariance structures for the random metric effects.**
(PDF)
