# Correction: Model-Based Assessment of Estuary Ecosystem Health Using the Latent Health Factor Index, with Application to the Richibucto Estuary

**DOI:** 10.1371/annotation/e5fb3d6a-143d-42a9-ad2e-ebfabd496dbd

**Published:** 2014-01-03

**Authors:** Grace S. Chiu, Margaret A. Wu, Lin Lu

Incorrect references occurred in the Introduction. In the second paragraph, the sentence that begins “Chiu and Guttorp” should reference “Chiu and Guttorp [15].”

Incorrect references occur in the Methods section. The fourth sentence in the first paragraph should read: “Observed alongside benthic fauna were abiotic properties of the estuary (Table 1 in [19]).”

Incorrect references occur in the Methods section below Equation 9. The correct sentence is: “Then, one can take advantage of existing MCMC software such as OpenBUGS [29] for straightforward implementation of the LHFI model, although in our experience, nontrivial hierarchical centering is essential to improve MCMC mixing [17,18].”

Incorrect references occur in the paragraph below Equation 8 of the Methods section. The corrected sentences are: "With a small dataset from 18 Richibucto sites each with only 2 to 3 replicate grab samples, a practical concern is that a general Σ may be only weakly identifiable depending on the complexity of the covariance structure (see [20] for a discussion on lack of identifiability in Bayesian inference). This issue was encountered in [17] when a fully unstructured Σ was assumed for a freshwater benthic dataset that also involved 18 sites with 3 replicates per site, but with nine metrics altogether.”

Incorrect references occur in the Results section. The last sentence of the third paragraph under “Simultaneously Significant Covariates in Two-Level Health Regression” should read: “To assess the model’s predictive ability for latent health, one could conduct a simulation study in which unobservable Hi values are generated then estimated, although such an approach for hierarchical models has its shortcomings [31] or requires intensive computations [32] that may be impractical.”

The incorrect data appears under the section “Identifying Drivers of Estuary Health” in the Methods section. The second paragraph should note the geographical domain of "(4km)x(3km)".

The incorrect symbol appears in Equation 8 in the Methods section. The second half of the equation should include a boldfaced Sigma. Please see the correct Equation 8 here: http://plosone.org/corrections/pone.0065697.e008.cn.tif

The incorrect symbol appears in Equation 12 in the Methods section. The χ symbol should be an x. Please see the correct Equation 12 here: http://plosone.org/corrections/pone.0065697.e012.cn.tif

The incorrect reference appears in the Figure 5 legend. The correct reference should be "Lu et al. [19]". In the paragraph above the Discussion section, this reference also appears incorrectly as “Lu et al. [20]” and should appear as “Lu et al. [19]”.

An error occurred in Table 5. The Description category for Model 5 should include the α symbol. The correct part of the equation is Cor(α). 

