# Supplementary material for: Model-Based Assessment of Estuary Ecosystem Health Using the Latent Health Factor Index, with Application to the Richibucto Estuary
Source: PLoS One. 2013 Jun 13;8(6):e65697. doi: 10.1371/journal.pone.0065697 (PMC3681865; doi:10.1371/journal.pone.0065697)
Supplement: Appendix S1 — Richibucto data collected and studied by Lu et al. [19] . (PDF) [file pone.0065697.s001.pdf]

Appendix S1 for "Model-Based Assessment of Estuary Ecosystem Health using the Latent Health Factor Index, with Application to the Richibucto Estuary" by Chiu et al.

Grace S. Chiu[1,*], Margaret A. Wu[2], Lin Lu[3]

[1] CSIRO Mathematics, Informatics and Statistics, Commonwealth Scientific and Industrial Research Organisation (CSIRO), Canberra, Australian Capital Territory, Australia

[2] Business Methods Survey Division, Statistics Canada, Ottawa, Ontario, Canada

[3] McGregor GeoScience, Bedford, Nova Scotia, Canada

[*] E-mail: grace.chiu@csiro.au

The Richibucto data collected and studied by Lu et al. [1] include benthic count data (Tables S1–S3 below) and abiotic covariates (Table 3 in the main text and Table 1 in [1]).

Table S1. Number of replicate grab samples at each Richibucto site and corresponding counts of benthic organisms for each replicate.

Site	Number of Replicates	Total Organism Counts		
		First Replicate	*Second Replicate*	*Third Replicate*
1	3	136	194	140
2	3	389	449	337
3	2	167	183	n/a
4	3	230	208	205
5	3	198	277	96
6	2	269	336	n/a
7	2	264	391	n/a
8	2	285	357	n/a
9	3	224	210	238
10	3	189	125	103
11	3	223	212	170
12	2	467	214	n/a
13	3	324	337	273
14	3	216	173	193
15	3	454	249	551
16	3	456	430	532
17	3	195	245	288
18	3	89	128	94

Table S2. AMBI abundance metric values for Richibucto sites. Roman numerals denote metric numbers—see Table 1 in the main text for metric definition.

Site	*First Replicate*					*Second replicate*					*Third replicate*				
	I	II	III	IV	V	I	II	III	IV	V	I	II	III	IV	V
1	2	11	55	61	7	0	17	104	65	8	1	13	69	53	4
2	3	121	109	152	4	8	139	146	156	0	8	108	81	140	0
3	20	9	76	56	6	17	18	88	57	3			*n/a*		
4	9	29	112	79	1	8	23	118	57	2	8	25	96	72	4
5	9	34	27	53	75	8	96	47	86	40	4	33	29	26	4
6	8	39	134	85	3	3	31	213	81	8			*n/a*		
7	15	121	77	50	1	37	163	112	74	5			*n/a*		
8	13	39	125	81	27	32	37	141	98	49			*n/a*		
9	23	63	50	78	10	23	62	62	63	0	16	45	69	102	6
10	19	30	76	58	6	9	21	25	61	9	10	9	23	57	4
11	21	46	68	83	5	17	49	57	77	12	26	23	50	62	9
12	53	116	52	246	0	79	30	82	22	1			*n/a*		
13	31	60	59	126	48	37	65	67	83	85	16	27	58	105	67
14	20	28	82	64	22	20	23	66	54	10	22	34	54	72	11
15	120	45	223	66	0	42	32	142	33	0	146	94	254	55	2
16	91	73	243	46	3	74	70	220	65	1	109	146	217	60	0
17	35	70	36	43	11	56	92	56	38	3	44	96	72	45	31
18	20	25	13	24	7	42	58	4	19	5	25	42	8	11	8

Table S3. ITI abundance metric values for Richibucto sites. Roman numerals denote metric numbers—see Table 2 in the main text for metric definition.

Site	*First Replicate*				*Second replicate*				*Third replicate*			
	I	II	III	IV	I	II	III	IV	I	II	III	IV
1	0	121	8	7	0	177	9	8	0	128	8	4
2	0	378	7	4	3	441	5	0	2	330	5	0
3	19	127	15	6	15	130	35	3		*n/a*		
4	5	191	33	1	1	184	21	2	1	176	24	4
5	0	117	6	75	0	230	7	40	0	89	3	4
6	6	244	16	3	5	314	9	8		*n/a*		
7	1	236	26	1	2	355	29	5		*n/a*		
8	7	225	26	27	16	261	31	49		*n/a*		
9	14	174	26	10	1	185	24	0	7	197	28	6
10	2	161	20	6	8	102	6	9	3	86	10	4
11	6	155	57	5	3	158	39	12	3	113	45	9
12	9	431	27	0	11	146	56	1		*n/a*		
13	32	194	49	49	24	195	33	85	18	160	28	67
14	34	123	37	22	32	108	23	10	24	130	28	11
15	16	325	60	53	27	174	27	21	46	368	100	37
16	48	331	61	16	39	317	44	30	43	386	69	34
17	7	156	21	11	19	191	31	4	14	224	18	32
18	2	72	7	8	7	105	11	5	4	76	6	8

References

1. Lu L, Grant J, Barrell J (2008) Macrofaunal spatial patterns in relationship to environmental variables in the Richibucto estuary, New Brunswick, Canada. Estuaries and Coasts 31: 994–1005.
