# Supplementary material for: Model-Based Assessment of Estuary Ecosystem Health Using the Latent Health Factor Index, with Application to the Richibucto Estuary
Source: PLoS One. 2013 Jun 13;8(6):e65697. doi: 10.1371/journal.pone.0065697 (PMC3681865; doi:10.1371/journal.pone.0065697)
Supplement: Appendix S2 — LHFI-A-I: integrating AMBI and ITI metrics. (PDF) [file pone.0065697.s002.pdf]

Appendix S2 for "Model-Based Assessment of Estuary Ecosystem Health using the Latent Health Factor Index, with Application to the Richibucto Estuary" by Chiu et al.

Grace S. Chiu[1,*], Margaret A. Wu[2], Lin Lu[3]

[1] CSIRO Mathematics, Informatics and Statistics, Commonwealth Scientific and Industrial Research Organisation (CSIRO), Canberra, Australian Capital Territory, Australia

[2] Business Methods Survey Division, Statistics Canada, Ottawa, Ontario, Canada

[3] McGregor GeoScience, Bedford, Nova Scotia, Canada

[*] E-mail: grace.chiu@csiro.au

The LHFI-A-I model is an extension of the preliminary LHFI-A model, where an extra component for ITI is constructed in the same fashion and with the same reasoning as for AMBI, then added to the existing LHFI-A model (Equations 1–7 in the main text) to form the LHFI-A-I model. We do so as follows.

Let m denote the dataset, where $m = A$ represents AMBI and $m = I$ represents ITI. Similar to the LHFI-A model, the ITI metrics are split into two groups: $s =$ "$-$" containing the third and fourth metrics which are, respectively, indifferent and negatively related to health; and $s =$ "$+$" containing the first and second metrics which are positively related to health. For $s =$ "$-$" the link function is inverted as in Equation 4. Since $s =$ "$-$" and $s =$ "$+$" have the same interpretation regardless of the value of m, we cross s with m to define an interaction. Thus, metric effects are nested in $s \times m$; that is, $\beta_{j(m\times s)}$ denotes the effect on the linear predictor ν due to the jth metric in the sth metric group and the mth dataset. For AMBI, we take the variance of the βs to be $\sigma^2_{\beta A}$ which is constant across j and s, as deemed reasonable from fitting the LHFI-A model. For ITI, however, we avoid the a priori assumption of constant variance for metric effects, and so we consider $\sigma^2_{j(I\times s)}$.

Next, to explain the difference among the dataset-metric combinations of $m \times s$, note that any crossed term in principle can be broken down into main effects and an interaction. Thus, we let

$$\beta_{j(m\times s)} = \gamma_m + \zeta_s + \xi_{m\times s} + \omega_{j(m\times s)}$$

where ζ_s, γ_m, and $\xi_{m\times s}$ represent the fixed effects of metric group, dataset, and their interaction, respectively, on metric effect β;[1] the mean-zero random error is $\omega_{j(m\times s)}$, and we take $\zeta_s \equiv 0$ due to aliasing with θ_s. Altogether, the LHFI-A-I model comprises

$$\{Y_{i1k\ell A+}, Y_{i2k\ell A+}, N_{ik\ell A} - \sum_{j=1}^{2} Y_{ijk\ell A+}\} | N_{ik\ell A}, p_{i1\ell A+}, p_{i2\ell A+}$$
$$\sim \text{Multinomial}(N_{ik\ell A}; p_{i1\ell A+}, p_{i2\ell A+}, 1 - \sum_{j=1}^{2} p_{ij\ell A+}),$$
$$\{Y_{i3k\ell A-}, Y_{i4k\ell A-}, Y_{i5k\ell A-}, N_{ik\ell A} - \sum_{j=3}^{5} Y_{ijk\ell A-}\} | N_{ik\ell A}, p_{i3\ell A-}, p_{i4\ell A-}, p_{i5\ell A-}$$
$$\sim \text{Multinomial}(N_{ik\ell A}; p_{i3\ell A-}, p_{i4\ell A-}, p_{i5\ell A-}, 1 - \sum_{j=3}^{5} p_{ij\ell A-}),$$
$$\nu_{ij\ell A+} = \log \frac{p_{ij\ell A+}}{1 - \sum_{j=1}^{2} p_{ij\ell A+}} \text{ for } j = 1, 2, \quad \nu_{ij\ell A-} = \log \frac{1 - \sum_{j=3}^{5} p_{ij\ell A-}}{p_{ij\ell A-}} \text{ for } j = 3, 4, 5,$$
$$\{Y_{i1k\ell I+}, Y_{i2k\ell I+}, N_{ik\ell I} - \sum_{j=1}^{2} Y_{ijk\ell I+}\} | N_{ik\ell I}, p_{i1\ell I+}, p_{i2\ell I+}$$
$$\sim \text{Multinomial}(N_{ik\ell I}; p_{i1\ell I+}, p_{i2\ell I+}, 1 - \sum_{j=1}^{2} p_{ij\ell I+}),$$
$$\{Y_{i3k\ell I-}, Y_{i4k\ell I-}, N_{ik\ell I} - \sum_{j=3}^{4} Y_{ijk\ell I-}\} | N_{ik\ell I}, p_{i3\ell I-}, p_{i4\ell I-}$$
$$\sim \text{Multinomial}(N_{ik\ell I}; p_{i3\ell I-}, p_{i4\ell I-}, 1 - \sum_{j=3}^{4} p_{ij\ell I-}),$$
$$\nu_{ij\ell I+} = \log \frac{p_{ij\ell I+}}{1 - \sum_{j=1}^{2} p_{ij\ell I+}} \text{ for } j = 1, 2, \quad \nu_{ij\ell I-} = \log \frac{1 - \sum_{j=3}^{4} p_{ij\ell I-}}{p_{ij\ell I-}} \text{ for } j = 3, 4,$$
$$\nu_{ij\ell ms} = H_{i(\ell)} + \theta_s + \beta_{j(m\times s)}, \quad H_{i(\ell)} = \alpha_0 + f(\boldsymbol{\alpha}, \boldsymbol{x}_{i(\ell)}) + \varepsilon_{i(\ell)},$$
$$\varepsilon_{i(\ell)} | \sigma^2_{H\ell} \stackrel{\text{ind}}{\sim} \text{N}(0, \sigma^2_{H\ell}), \quad \beta_{j(m\times s)} = \gamma_m + \xi_{m\times s} + \omega_{j(m\times s)}, \quad \theta_+ = \gamma_A = \xi_{A+} = \xi_{A-} = \xi_{I+} = 0,$$
$$\omega_{j(A\times s)} | \sigma^2_{\beta A} \stackrel{\text{iid}}{\sim} \text{Normal}(0, \sigma^2_{\beta A}), \quad \omega_{j(I\times s)} | \sigma^2_{j(I\times s)} \stackrel{\text{ind}}{\sim} \text{Normal}(0, \sigma^2_{j(I\times s)}).$$

[1]Unlike for LHFI-A, β under this formulation of LHFI-A-I is not a mean-zero error term, but this does not change the intuition of the model as βs are nuisance parameters. Alternatively, if a mean-zero β were desired, then γ_m and $\xi_{m\times s}$ could be placed in the model hierarchy alongside θ_s where they would all directly explain ν instead of β.
