# Supplementary material for: Model-Based Assessment of Estuary Ecosystem Health Using the Latent Health Factor Index, with Application to the Richibucto Estuary
Source: PLoS One. 2013 Jun 13;8(6):e65697. doi: 10.1371/journal.pone.0065697 (PMC3681865; doi:10.1371/journal.pone.0065697)
Supplement: Appendix S3 — Some covariance structures for the random metric effects. (PDF) [file pone.0065697.s003.pdf]

Appendix S3 for "Model-Based Assessment of Estuary Ecosystem Health using the Latent Health Factor Index, with Application to the Richibucto Estuary" by Chiu et al.

Grace S. Chiu[1,*], Margaret A. Wu[2], Lin Lu[3]

[1] CSIRO Mathematics, Informatics and Statistics, Commonwealth Scientific and Industrial Research Organisation (CSIRO), Canberra, Australian Capital Territory, Australia

[2] Business Methods Survey Division, Statistics Canada, Ottawa, Ontario, Canada

[3] McGregor GeoScience, Bedford, Nova Scotia, Canada

[*] E-mail: grace.chiu@csiro.au

For a structured $\boldsymbol{\Sigma}$, consider the following.

Since the value of i determines ℓ and that of j determines s, drop the subscripts ℓ and s from p and ν in Equations 3–5 of the main text. Then, given site i, let $\boldsymbol{\Sigma}^\nu$ denote the 5×5 covariance matrix whose (j, j')th element is $\Sigma^\nu_{jj'} = \text{Cov}(\nu_{ij}, \nu_{ij'})$ which does not depend on i. In the frequentist context, one can show that $\boldsymbol{\beta}$ has covariance matrix

$$\boldsymbol{\Sigma} = \boldsymbol{\Sigma}^\nu - \sigma_H^2 \boldsymbol{J}_{55} \tag{S1}$$

where $\boldsymbol{J}_{dd'}$ is a $d \times d'$ matrix of 1s. Furthermore, partition $\boldsymbol{\Sigma}^\nu$ into "+", "−" and "±" blocks accordingly. To limit the overall complexity of $\boldsymbol{\Sigma}^\nu$ (and hence, $\boldsymbol{\Sigma}$), assume that the association between the vectors of ν_+s and ν_-s is adequately accounted for by the fixed effect θ_s in Equation 5, and consequently $\boldsymbol{\Sigma}^\nu_\pm$ is a matrix of 0s. However, allow $\boldsymbol{\Sigma}^\nu_+$ and $\boldsymbol{\Sigma}^\nu_-$ to be unstructured. Thus, one could consider a formulation of $\boldsymbol{\Sigma}$ whose general form mimics Equation S1, with unstructured positive definite blocks along the diagonal, and constant off-diagonal matrices:

$$\boldsymbol{\Sigma}_+(\boldsymbol{\Lambda}_+, \varsigma) \equiv \boldsymbol{\Lambda}_+ + \varsigma \boldsymbol{J}_{22}\,, \quad \boldsymbol{\Sigma}_-(\boldsymbol{\Lambda}_-, \varsigma) \equiv \boldsymbol{\Lambda}_- + \varsigma \boldsymbol{J}_{33}\,, \quad \boldsymbol{\Sigma}_\pm(\varsigma) \equiv \varsigma \boldsymbol{J}_{23} \tag{S2}$$

$$\boldsymbol{\Lambda}_+ \sim \text{IW}_2\,, \quad \boldsymbol{\Lambda}_- \sim \text{IW}_3 \tag{S3}$$

$$\varsigma | \boldsymbol{\Lambda}_+, \boldsymbol{\Lambda}_- \sim \text{N}(0, 100) \text{ subject to } \boldsymbol{\Sigma}(\boldsymbol{\Lambda}_+, \boldsymbol{\Lambda}_-, \varsigma) \text{ being positive definite} \tag{S4}$$

In general, the structure from Equations S2–S4 is a reasonable compromise between taking a fully unstructured $\boldsymbol{\Sigma} \sim \text{IW}_d$ (with many parameters) and the block diagonal structure of Equation 9 in the main text (potentially too simplistic). However, note that the prior in Equation S4 itself is not a true Gaussian

distribution due to the positive definite constraint. Thus, MCMC implementation for the LHFI model involving Equations S2–S4 would be nontrivial, whether using OpenBUGS or another programming language.
